# Long intergenic non-protein coding RNA 152 promotes multiple myeloma progression by negatively regulating microRNA-497

**DOI:** 10.3892/or.2021.8239

**Published:** 2021-12-03

**Authors:** Tianhua Yu, Zhihua Xu, Xuanhe Zhang, Lan Men, Haiying Nie

Oncol Rep 40: 3763-3771, 2018; DOI: 10.3892/or.2018.6721

Following the publication of this paper, an interested reader drew the authors’ attention to the fact that several of the tumour images shown in Fig. 6B were strikingly similar to images that had appeared in another paper by different authors published at around the same time. The authors also admitted that the *in vivo* experiments reported in this study had been performed by a third party. Consequently, owing to a lack of confidence in the presented data, the authors requested that this paper be retracted from the Journal.

The Editor was in agreement that the paper should be retracted. All authors agree with the retraction of this article, and the Editor apologizes to the readership for any inconvenience caused.

